# Differential renal proteomics analysis in a novel rat model of iodinated contrast-induced acute kidney injury

**DOI:** 10.1080/0886022X.2023.2178821

**Published:** 2023-02-16

**Authors:** Ying-Hao Deng, Xiu-Fen Wang, Xi Wu, Ping Yan, Qian Liu, Ting Wu, Shao-Bin Duan

**Affiliations:** Department of Nephrology, Hunan Key Laboratory of Kidney Disease and Blood Purification, The Second Xiangya Hospital of Central South University Changsha, Hunan, P.R. China

**Keywords:** Contrast media, toxicity, acute kidney injury, proteomics, tandem mass tag, parallel reaction monitoring

## Abstract

Contrast-induced acute kidney injury (CI-AKI), which occurs after the use of iodinated contrast media, has become the third leading cause of hospital-acquired acute kidney injury (AKI). It is associated with prolonged hospitalization and increased risks of end-stage renal disease and mortality. The pathogenesis of CI-AKI is unclear and effective treatments are lacking. By comparing different post-nephrectomy times and dehydration times, we constructed a new, short-course CI-AKI model using dehydration for 24 h two weeks after unilateral nephrectomy. We found that the low-osmolality contrast media iohexol caused more severe renal function decline, renal morphological damage, and mitochondrial ultrastructural alterations compared to the iso-osmolality contrast media iodixanol. The shotgun proteomics based on Tandem Mass Tag (TMT) was used to conduct proteomics research on renal tissue in the new CI-AKI model, and 604 distinct proteins were identified, mainly involving complement and coagulation cascade, COVID-19, PPAR signalling pathway, mineral absorption, cholesterol metabolism, ferroptosis, staphylococcus aureus infection, systemic lupus erythematosus, folate biosynthesis, and proximal tubule bicarbonate reclamation. Then, using parallel reaction monitoring (PRM), we validate 16 candidate proteins, of which five were novel candidates (Serpina1, Apoa1, F2, Plg, Hrg) previously unrelated to AKI and associated with an acute response as well as fibrinolysis. The pathway analysis and 16 candidate proteins may help to discover new mechanisms in the pathogenesis of CI-AKI, allowing for early diagnosis and outcome prediction.

## Introduction

Contrast-induced acute kidney injury (CI-AKI) is an acute kidney injury caused by intravascular administration of contrast media, excluding other causes. It is defined as an increase of ≥0.3 mg/dl (26.5 μmol/l) in serum creatinine (Scr) level from baseline within 48 h of exposure to contrast media or at least a 1.5-fold increase from baseline within 7 days [1]. In recent years, CI-AKI has become a global public health problem, with a prevalence of 0.6% to 2.3% in the general population without underlying disease and can exceed 20% in high-risk groups with chronic underlying disease [2–4]. Furthermore, CI-AKI is associated with increased risks of persistent renal impairment that may progress to chronic kidney disease, end-stage renal disease, or even death [5–8].

To date, the pathogenesis of CI-AKI remains unclear. It is generally believed that the pathogenesis of CI-AKI is due to altered hemodynamics, direct toxic effects, inflammation, and generation of reactive oxygen species [9–16]. Some clinical studies indicated that hydration and statins can increase endothelial nitric oxide production, and improve vascular tone, thereby producing anti-inflammatory and antioxidant effects, which may help prevent the development of CI-AKI [17]. However, effective treatments for CI-AKI are still lacking. Therefore, it is significant to explore the new mechanism of CI-AKI to identify therapeutic targets and postpone the progression of CI-AKI to chronic kidney disease.

Proteomics is a technique for studying how proteins interact with each other and the roles they play in an organism. It provides an accurate description of cellular function under physiological and pathological conditions and is essential for early diagnosis, prognosis, and monitoring of disease progression. Previous studies have reported the differentially expressed urine proteins in CI-AKI patients [18,19], but few studies have looked into differentially expressed protein profiling in CI-AKI renal tissues.

Current CI-AKI rat models have some limitations, including the inability to simulate the pathophysiological changes seen in CI-AKI patients, low levels of creatinine elevation, and long modeling time [20–23]. In this study, we aimed to develop a new CI-AKI rat model and identify potential CI-AKI therapeutic targets using TMT technology and parallel reaction monitoring validation.

## Materials and methods

Sixty Sprague Dawley (SD) male rats, aged 5 weeks, weighing about 180 g, were provided by Hunan Slaughter Laboratory Animal Technology Co. All rats were housed in the Medical Animal Experiment Center of Central South University for 5 days in the same animal room, with temperature control at 20–22 °C, 12h cyclic light, and an unrestricted diet. Experimental operations followed the Regulations on the Administration of Laboratory Animals of the National Science and Technology Commission. The nonionic low-osmolar contrast medium iohexol (350; 300 mg iodine/mL; GE Healthcare, Shanghai, China) was used. CI-AKI is defined as an increase in Scr ≥50% from the baseline value within 48 h in Kidney Disease Improving Global Outcomes (KDIGO) [1].

### Establishment of the new CI-AKI rat model

This study was divided into three parts. The first part investigated the optimal time for intervention after left nephrectomy to establish the CI-AKI rat model.

Twenty-four rats were enrolled and randomly assigned to six experimental groups (*n* = 4 per group): (1) one-week post-surgery +24 h dehydration + saline group: 24 h dehydration was performed one week after surgery, followed by intraperitoneal injection of furosemide (10 mL/kg), and 30 min later saline (15 mL/kg) *via* the tail vein; (2) two-week post-surgery +24 h dehydration + saline group: 24 h dehydration was performed two weeks after surgery, followed by intraperitoneal injection of furosemide (10 mL/kg), and 30 min later saline (15 mL/kg) *via* the tail vein; (3) three-week post-surgery +24 h dehydration + saline group: 24 h dehydration was performed three weeks after surgery, followed by intraperitoneal injection of furosemide (10 mL/kg), and 30 min later saline (15 mL/kg) *via* the tail vein; (4) one-week post-surgery +24 h dehydration + iohexol group: 24 h dehydration was performed one week after surgery, followed by intraperitoneal injection of furosemide (10 mL/kg), and 30 min later iohexol (15 mL/kg) *via* the tail vein; (5) two-week post-surgery +24 h dehydration + iohexol group: 24 h dehydration was performed two weeks after surgery, followed by intraperitoneal injection of furosemide (10 mL/kg), and 30 min later iohexol (15 mL/kg) *via* the tail vein; (6) three-week post-surgery + 24 h dehydration + iohexol group: 24 h dehydration was performed three weeks after surgery, followed by intraperitoneal injection of furosemide (10 mL/kg), and 30 min later iohexol (15 mL/kg) *via* the tail vein; The doses of contrast media and furosemide were selected as previously described [21]. The left nephrectomy was performed under general anesthesia with pentobarbital sodium. In this study, the most significant increase in serum creatinine was observed in the rats reinjected with iohexol two weeks after surgery. Therefore, two weeks after the surgery was chosen as the preferred time for part two and part three.

**Figure 1.**
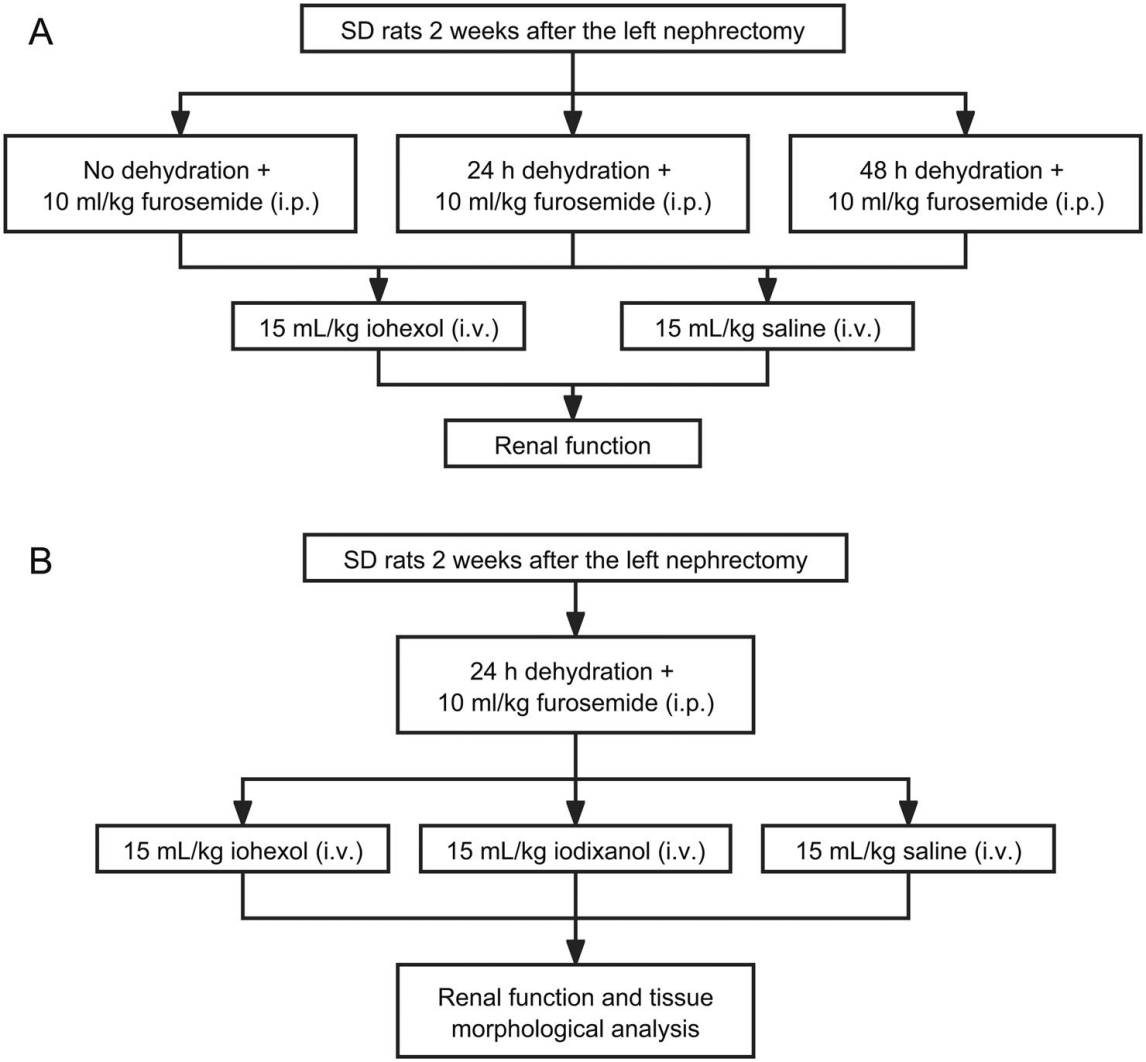
Animal study design. i.v, intravenous. i.p, intraperitoneal.

In the second part, multiple dehydration pretreatment plans were investigated to find the optimal precondition procedure (Figure 1(A)). Twenty-four rats with similar renal function 2 weeks after the surgery were enrolled and randomly assigned to six experimental groups (*n* = 4 in each group): (1) saline group; (2) 24 h dehydration + saline group; (3) 48 h dehydration + saline group; (4) iohexol group; (5) 24 h dehydration + iohexol group; (6) 48 h dehydration + iohexol group. It was found that administration of iohexol (15 mL/kg) along with 24 h dehydration could cause CI-AKI.

The final phase involved using the novel model to compare the toxic effects of iodixanol and iohexol on the kidneys. For this purpose, fifteen rats with comparable renal function two weeks after surgery were enlisted and randomly allocated to one of three experimental groups (*n* = 5 in each group) (Figure 1(B)). All rats were dehydrated for 24 h before tail vein injection of a contrast medium (3.2 g I/kg) and/or 0.9% saline. (1) rats in the control group were injected with 0.9% saline at a dose of 15 mL/kg; (2) rats in the iodixanol group, iodixanol (320 mg iodine/mL) 15 mL/kg; (3) rats in the iohexol group, a combination of iohexol (350 mg iodine/mL) 6 mL/kg and iohexol (300 mg iodine/mL) 9 mL/kg to match the iodine content of iodixanol (320 mg iodine/mL).

### Collection of blood samples and kidney tissues

Blood samples were collected from the rats two days before surgery (as baseline) and 24 h after the contrast or saline injection (as end), and serum creatinine and blood urea nitrogen (BUN) levels were measured as well. The kidney tissues were collected 24 h after the contrast or saline injection.

### Renal tissue morphological analysis

Following quick isolation, kidney tissues were fixed with 4% formaldehyde and embedded in paraffin or fixed in 2.5% glutaraldehyde as required. To observe pathological alterations, histological staining was carried out using hematoxylin, eosin (HE) and periodic acid-Schiff (PAS). We chose 10 high-magnification (×200) fields of the cortex and outer stripe of the outer medulla at random for semiquantitative analysis of the frequency and severity of renal lesions. On a semiquantitative scale, the specimens were scored based on the extent of foamy degeneration and tubular cell detachment: no injury (0), mild: 25% (1), moderate: 50% (2), severe: 75% (3), and very severe: >75% (4) [24]. A transmission electron microscope from Hitachi, model H7700, was used to examine the renal cells’ ultrastructure.

### Sample preparation for mass spectrometry analysis

Three rats from the iohexol group and saline control group in the new model were randomly selected respectively, and anesthetized using sodium pentobarbital intraperitoneally. The abdomen was opened and perfused *via* saline and kidney tissues were retained, fresh-frozen in liquid nitrogen and stored at −80 °C in a freezer. 100 μg of protein was taken from each specimen and enzymatically digested using a filter-assisted sample preparation enzyme digestion method at 37 °C under the action of trypsin. Analysis was performed by nano-liquid chromatography-tandem mass spectrometry using an EASY-nLC (Thermo Fisher Scientific) 1,000 pump. The peptides were graded by high pH reversed-phase HPLC and loaded onto a C18 column in buffer A. (Buffer A: aqueous solution of 0.1% formic acid, Buffer B: acetonitrile solution of 0.1% formic acid). The gradient settings for peptide separation: 7%∼23% B-phase 0–36 min; 23%∼32% B-phase 36–52 min; 32%∼80% B-phase 52-56 min; 80% B-phase 56-60 min. The peptides were separated by the UHPLC system and injected into the NSI ion source for ionization.

### Liquid chromatography-mass spectrometry analysis and data processing

The peptide signals were analyzed using Q-Exactive™ HF-X mass spectrometry (Thermo Fisher Scientific). The mass range of the acquired data was 350–1600 m/z with a resolution of 120,000, the starting point of the secondary mass spectrometry scan range was 100 m/z, and the resolution of the second scan was 15,000. The data acquisition mode was selected as a data-dependent scan. The automatic gain control was set to 1 × 10^5^, the signal threshold was set to 8.3 × 10^4^ ions/s, the maximum injection time was set to 60 ms, and the dynamic exclusion time of the tandem mass spectrometry scan was set to 30 s. The output data were database searched using Maxquant (v1.6.15.0), and the UniProt database was selected to identify the peptides and obtain the number of peptides and the relative quantitative values of each protein.

### Bioinformatics

The R language packages “org.Rn.eg.db”, “clusterProfiler”" and “enrichplot” were used for Gene Ontology (GO) analysis of differential proteins, and the R language package “pathview” was also used for Kyoto Encyclopedia of Genes and Genomes (KEGG) pathway enrichment analysis.

Since proteins rarely work alone, it is necessary to study the interactions between proteins. After comparing the differentially expressed proteins with the STRING (v.11.5) protein-protein interaction (PPI) network interaction database, PPI networks with scores > 0.7 were selected and visualized by Cytoscape software. A plug-in called ClusterOne for Cytoscape was then used to filter the significant modules in the PPI networks [25].

Hub genes are a small number of important nodes in the PPI network that have more connections. The top 15 hub genes in the PPI network were identified by applying *cytoHubba*, a module of Cytoscape software [26]. Based on the *GeneMANIA* plugin in Cytoscape, the PPI networks of these 15 hub genes were constructed and visualized [27]. Finally, the module *ClueGo* was used to analyze the GO biological process of these 15 hub genes [28].

### Verification of protein expression levels by parallel reaction monitoring (PRM)

Protein extraction, trypsin digestion, and LC-MS/MS analysis were performed as described above. The resulting MS data were processed using Skyline (v.3.6). Peptide settings: enzyme was set as Trypsin [KR/P], Max missed cleavage set as 2. The peptide length was set as 8-25, Variable modification was set as Carbamidomethyl on Cys and oxidation on Met, and max variable modifications were set as 3. Transition settings: precursor charges were set as 2, 3, ion charges were set as 1, 2, and ion types were set as b, y, p. The product ions were set from ion 3 to the last ion, the ion match tolerance was set as 0.02 Da.

### Statistical analysis

Statistical analysis was performed using SPSS 22.0 statistical software. T-test for independent samples was used for protein difference comparisons between the CI-AKI model and the saline control group. Proteins with a differential expression fold change (FC) greater than 1.3-fold and statistically significant were defined as differentially expressed proteins. *p* < 0.05 was considered statistically significant.

## Results

### Establishment of the new CI-AKI rat model

**Figure 2.**
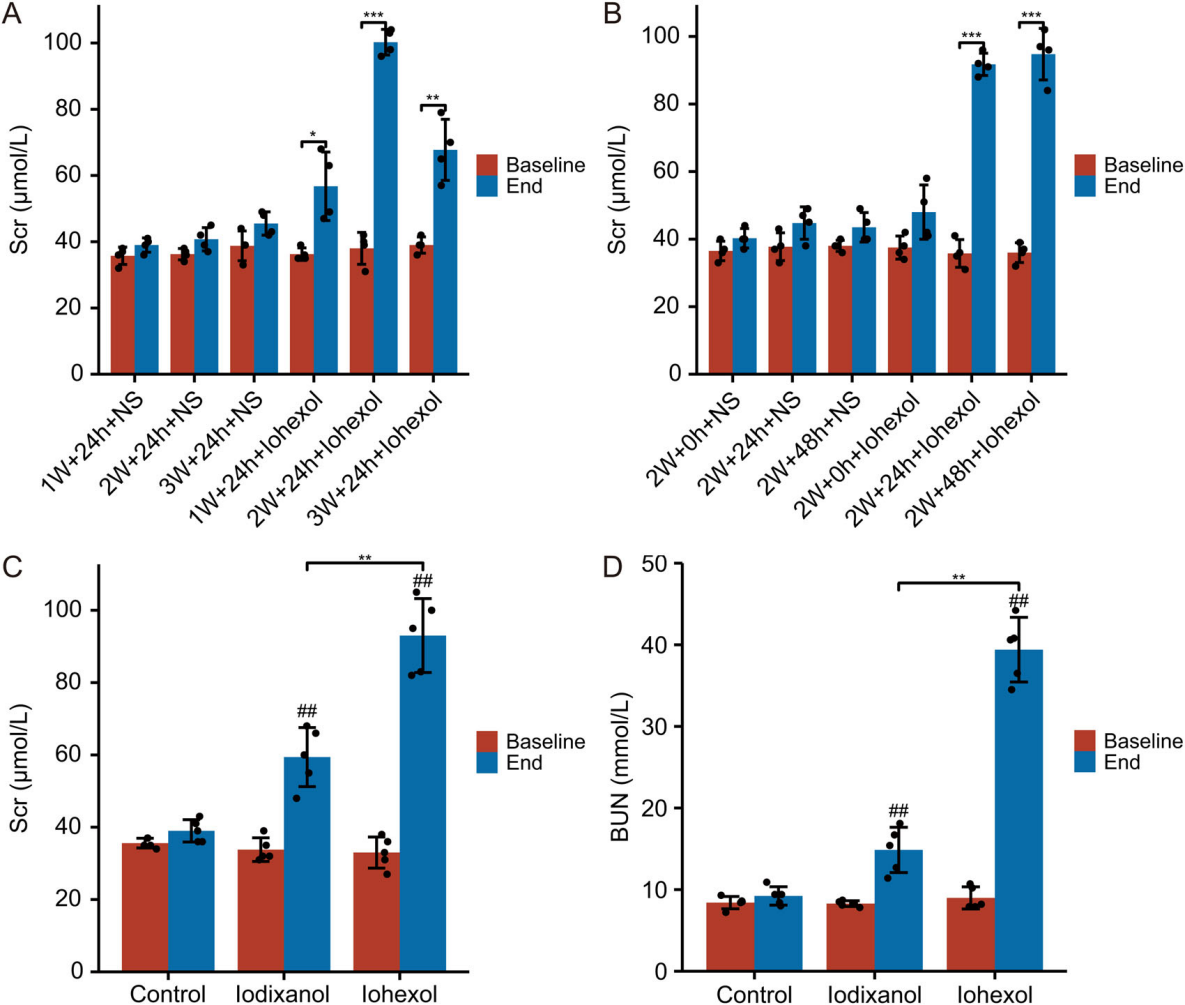
Data from animal studies. (**A, B).** Animals in the 2 W + 24H + iohexol group exhibited the most significant deterioration in renal function at 24 h post-injection compared to other groups of different post-surgical weeks (**A**) or various dehydration times (**B**); ***p < 0.001, **p < 0.01 and *p < 0.05 endpoint versus baseline in every group; *n* = 4. (**C, D).** In the novel CI-AKI rat model, iohexol induces a more significant reduction of renal function than iodixanol. Changes in the levels of (**C**) Scr, (**D**) BUN, 24 h after intravenous injection of saline, iohexol, or iodixanol. (##*p* < 0.01 vs. group control, **p < 0.01 vs. group iodixanol; *n* = 5).

Serum creatinine was significantly increased after iohexol injection at 1, 2, and 3 weeks after left nephrectomy, respectively, with the 2-week postoperative group showing the most significant increase in serum creatinine levels compared to baseline values (*p* < 0.001), whereas there was no significant change in serum creatinine before and after intervention in all saline groups (*p* > 0.05) (Figure 2(A)). In the rats two weeks after surgery, significant increases in Scr levels were observed 24 h after iohexol injection in both the 24 h dehydration + iohexol group and the 48 h dehydration + iohexol group compared to baseline levels (*p* < 0.001). There were no statistical differences between baseline and endpoint Scr levels in the saline, 24h dehydration + saline, 48h dehydration + saline, and iohexol groups (Figure 2(B)). To sum up, a novel CI-AKI rat model was successfully established by dehydration for 24h after 2 weeks of left nephrectomy, followed by intraperitoneal injection of furosemide (10 mL/kg) and iohexol administration *via* tail vein (15 mL/kg).

### Iohexol caused a more severe decrease in renal function, renal morphological damage and mitochondrial ultrastructural alterations compare to iodixanol

**Figure 3.**
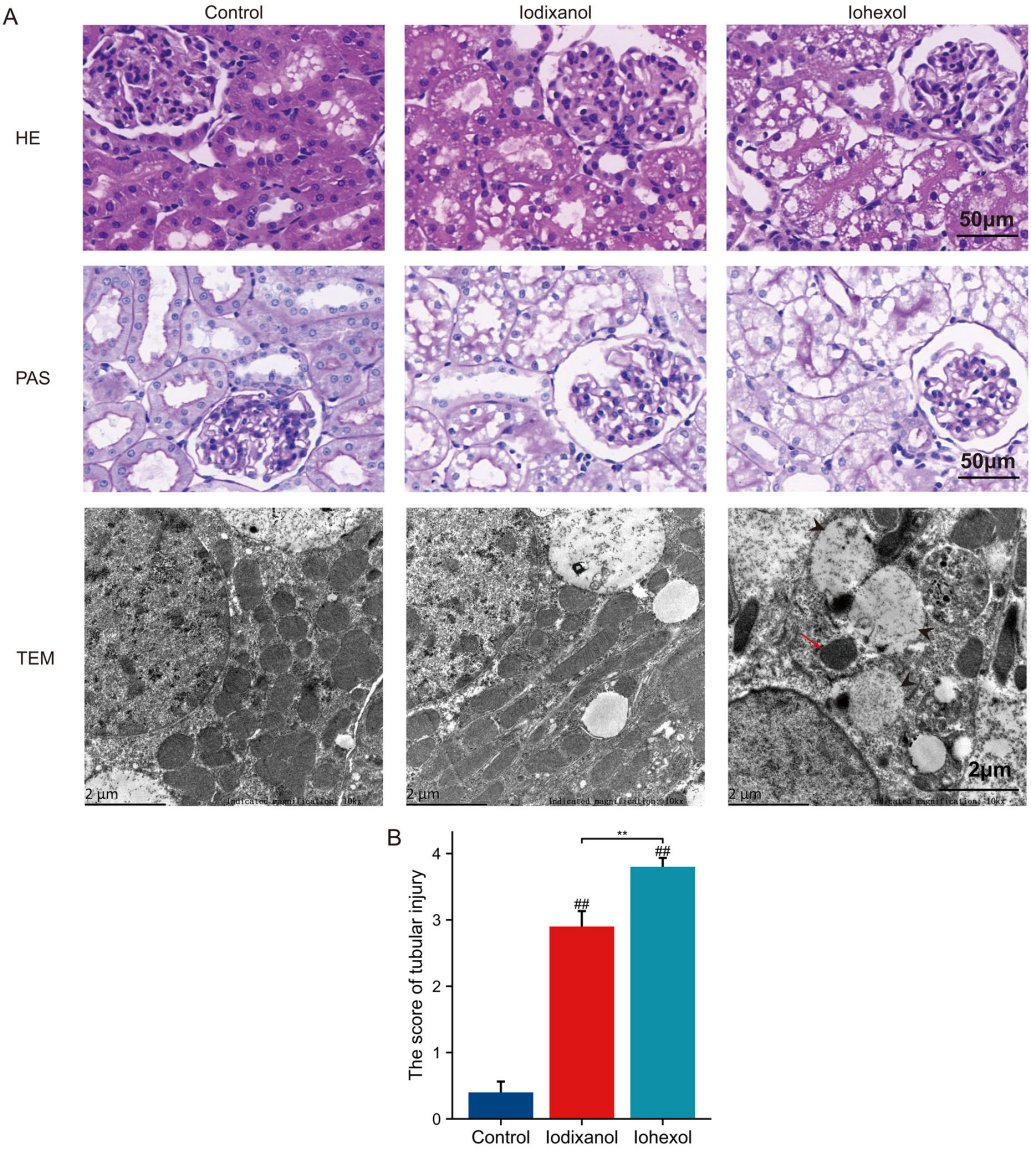
In the novel CI-AKI rat model, iodixanol induces more severe morphological damage than iodixanol. (**A)** Hematoxylin and eosin (HE) stain, scale bar, 50 μm; Periodic acid-Schiff (PAS) staining, scale bar, 50 μm; Transmission Electron Microscope (TEM), scale bars, 2 μm, representative photomicrographs of mitochondrial reduction, mitochondrial swelling, loss of cristae (red arrow), and vacuolization in the matrix (black triangle arrow) were observed in iohexol groups. (**B)** Quantitative analysis of histologic scoring. (^##^*p* < 0.01 vs. group control, **p < 0.01 vs. group iodixanol).

As shown in Figure 2(C and D), the levels of Scr and BUN were significantly higher in the iohexol and iodixanol groups compared to the control group (*p* < 0.01). At 24 h post-injection, Scr and BUN levels were noticeably greater in the iohexol group compared to the iodixanol group (*p* < 0.01). As shown by HE staining and PAS staining in Figure 3, the renal tubular epithelial cells in the iodixanol group exhibited swelling and focal vacuolar degeneration, while the cells in the iohexol group were more severely swollen with extensive vacuolar degeneration and brush border membrane detachment. Transmission electron microscopy showed that the mitochondria, endoplasmic reticulum, and nuclei of renal tubular epithelial cells in the control group had normal morphology; the mitochondria in the iodixanol group had basically normal morphology with clear mitochondrial cristae and focal vacuolar degeneration; the mitochondria in the iohexol group showed reduced mitochondria, mitochondrial swelling, loss of cristae, and extensive vacuolar degeneration. In summary, the iso-osmolality contrast agent iodixanol has lower nephrotoxicity than the low-osmolality contrast agent iohexol.

### Identification and screening of differentially expressed proteins from proteomics of rat kidney tissues

**Figure 4.**
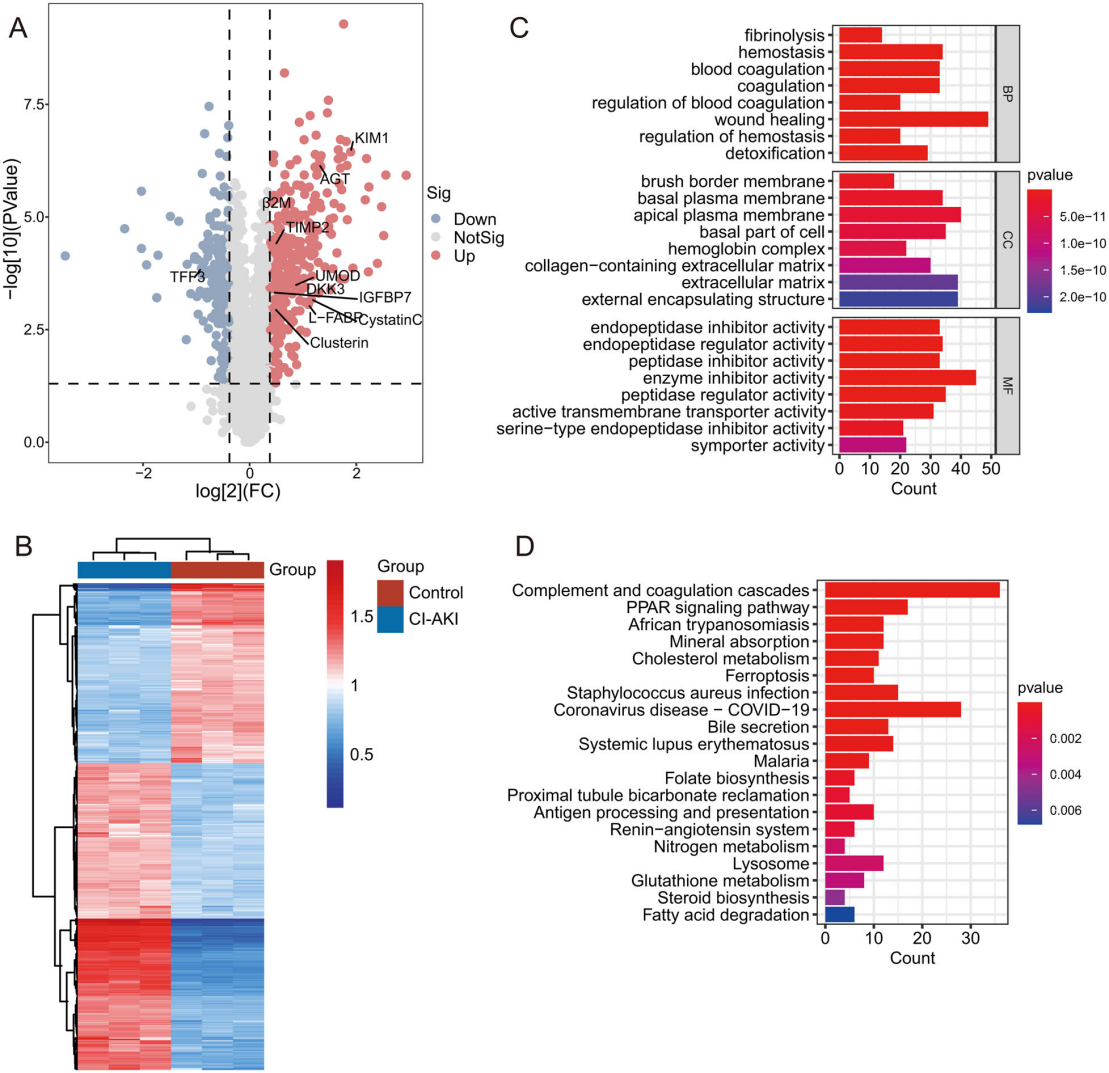
Results of Tandem Mass Tag proteomics analysis. (**A)** Volcano plot for 604 differentially expressed genes. (**B)** Heatmap representation of differentially expressed genes. (**C)** The GO enrichment analysis of 604 differentially expressed genes (top 8 were listed). (**D)** The KEGG enrichment analysis of 604 differentially expressed genes (top 20 were listed). GO, Gene Ontology; KEGG, Kyoto Encyclopedia of Genes and Genomes.

A total of 6546 proteins were successfully identified, among which 5498 proteins could be quantified. Then, 604 differentially expressed proteins were obtained by screening according to the differential fold (FC ≥ 1.30 or ≤0.77) and statistical significance (*p* < 0.05) criteria. A total of 381 proteins were up-regulated, and 223 were down-regulated in the iohexol CI-AKI group compared with the control group, with Table 1 presenting the 20 most significantly up-regulated proteins and Table 2 displaying the 20 most significantly down-regulated proteins, including 11 traditional and novel biomarkers of kidney injury that have been reported in reviews: Cystatin-C (Cst3), Beta-2-microglobulin (β2M), Kidney injury molecule 1 (Kim1/Havcr1), Metalloproteinase inhibitor 2 (Timp2), Insulin-like growth factor binding protein 7 (Igfbp7), Liver-type fatty acid-binding protein (L-Fabp/Fabp1), Angiotensinogen (Agt), Uromodulin (Umod), Dickkopf WNT-signaling pathway inhibitor 3 (Dkk3), Trefoil factor 3 (Tff3), and Clusterin (Clu) (Figure 4(A)) [29,30]. The heat map of 604 differentially expressed proteins is shown in Figure 4(B).

**Table 1.** Twenty proteins significantly up-regulated in CI-AKI renal tissues.

Accession	Protein Description	Gene	Average Fold Change (CI-AKI/Control)	*p*-Value
P01946	Hemoglobin subunit alpha-1/2	Hba1	7.652	<0.01
Q62669	Globin a1	LOC103694855	5.872	<0.01
O88752	Epsilon 1 globin	Hbe1	5.71	<0.01
A0A0G2JSV6	Globin c2	Hba-a2	5.557	<0.01
D4A7N7	Tetratricopeptide repeat domain 39D	Ttc39d	5.263	<0.01
P01048	T-kininogen 1	Map1	4.713	<0.01
P70668	Guanylate cyclase activator 2B	Guca2b	4.68	<0.01
P02091	Hemoglobin subunit beta-1	Hbb	4.567	<0.01
Q63910	Alpha globin	Hba-a3	4.492	<0.01
P04466	Myosin regulatory light chain 11	Mylpf	3.825	<0.01
G3V6W3	Kidney injury molecule 1	Havcr1/KIM1	3.72	<0.01
P02680	Fibrinogen gamma chain	Fgg	3.559	<0.01
A0A0G2JVA8	Type II keratin Kb15	Kb15	3.544	<0.01
A0A0H2UHI5	Serine protease inhibitor A3N	Serpina3n	3.506	<0.01
A0A0G2JTR6	Polyamine-modulated factor 1-binding protein 1	Pmfbp1	3.438	<0.01
P14480	Fibrinogen beta chain	Fgb	3.391	<0.01
P14046	Alpha-1-inhibitor 3	A1i3	3.356	<0.01
F7EUK4	Kininogen-1	Kng2	3.307	<0.01
D4A194	Centromere protein J	Cenpj	3.277	<0.01
Q6IRS6	Fetub protein	Fetub	3.258	<0.01

CI-AKI: Contrast-induced acute kidney injury.

**Table 2.** Twenty proteins significantly down-regulated in CI-AKI renal tissues.

Accession	Protein Description	Gene	Average Fold Change (CI-AKI/Control)	*p-*Value
A0A0G2JVB3	Trafficking regulator of GLUT4 1	Trarg1	0.091	<0.01
A0A0G2JTP6	Perilipin	Plin1	0.197	<0.01
A0A0G2JSK1	RCG20603	Serpina3c	0.245	<0.01
D3ZK35	Ciliogenesis and planar polarity effector 1	Cplane1	0.245	<0.01
P50170	Retinol dehydrogenase 16	Rdh16	0.262	<0.01
Q4TU71	Reproductive homeobox on X chromosome 12	Rhox12	0.299	<0.01
D3ZZ76	Uroplakin 3 A	Upk3a	0.304	<0.01
P04633	Mitochondrial brown fat uncoupling protein 1	Ucp1	0.357	<0.01
Q5XFV4	Adipocyte-type fatty acid-binding protein	Fabp4	0.398	<0.01
D4A2U6	LOC362424	Tmem72	0.439	<0.01
Q08290	Calponin-1	Cnn1	0.444	<0.01
A0A0G2K7V6	Sodium-dependent phosphate transport protein 2 C	Slc34a3	0.465	<0.01
A0A0G2JWZ2	Membrane primary amine oxidase	Aoc3	0.477	<0.01
F1LRQ7	Solute carrier organic anion transporter family member	Slc21a4	0.488	<0.01
G3V822	Carboxylic ester hydrolase	Ces1d	0.49	<0.01
Q01129	Decorin	Dcn	0.504	<0.01
D3ZZT9	Collagen type XIV alpha 1 chain	Col14a1	0.508	<0.01
F1LUV9	Neural cell adhesion molecule 1	Ncam1	0.512	<0.01
P46720	Solute carrier organic anion transporter family member 1A1	Slco1a1	0.526	<0.01
Q03191	Trefoil factor 3	Tff3	0.534	<0.01

CI-AKI: Contrast-induced acute kidney injury.

### GO enrichment analysis and KEGG enrichment analysis of differentially expressed proteins

In biological processes, differentially expressed proteins are associated with wound healing, hemostasis, coagulation, and fibrinolysis, while in terms of molecular functions, differentially expressed proteins have roles in (endo) peptidase inhibitors and active transmembrane transporter activity. As far as cellular components are concerned, differentially expressed proteins are associated with the apical plasma membrane, extracellular matrix, cellular substrate and plasma membrane, and hemoglobin complex (Figure 4(C)).

KEGG pathway enrichment analysis revealed that differentially expressed proteins were mainly involved in complement and coagulation cascade, COVID-19, PPAR signalling pathway, mineral absorption, cholesterol metabolism, ferroptosis, staphylococcus aureus infection, bile secretion, systemic lupus erythematosus, folate biosynthesis, and proximal tubule bicarbonate reclamation (Figure 4(D)).

### Protein-protein interaction network analysis

**Figure 5.**
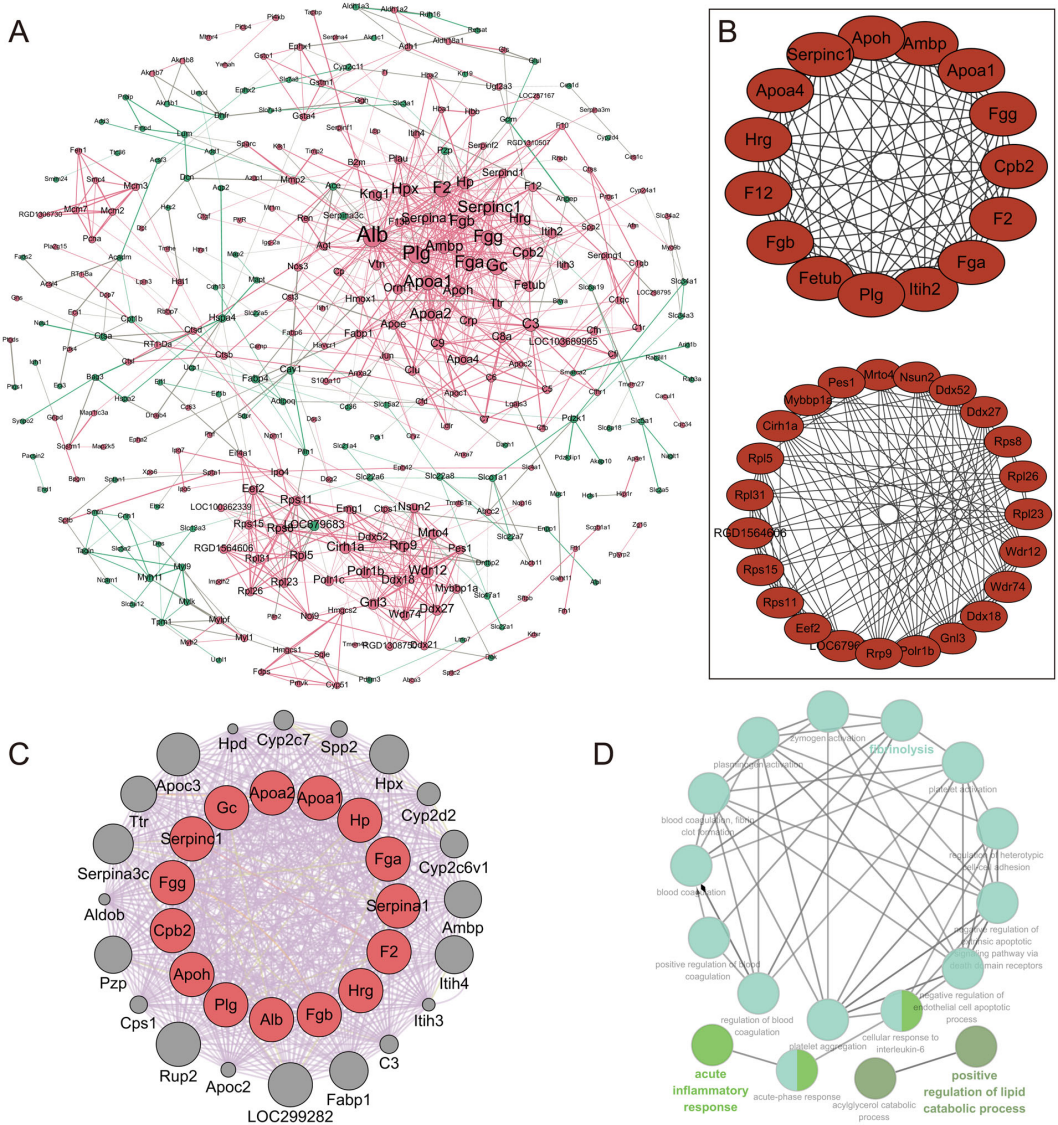
Protein-protein interaction network analysis. (**A)** Protein-protein interaction network of the 604 differentially expressed genes with a score >0.7; Disconnected nodes were hiding in the network; Red nodes represent up-regulated proteins, and the green represents down-regulated proteins. (**B)** The PPI network of two significant modules selected by *ClusterOne*. (**C)** The top 15 nodes were extracted as hub genes using module *cytoHubba*, displayed by the *GeneMANIA* plug-in in Cytoscape. (**D**) The interaction network of GO terms generated by the Cytoscape plug-in *ClueGO*. The significant term of each group is highlighted.

In the STRING database of PPI networks, there are 604 differentially expressed proteins with 562 nodes and 983 edges (Figure 5(A)). Figure 5(B) shows the results of the two most vital modules screened by *ClusterOne*. The top 15 nodes in module *cytoHubba* were extracted using the EPC, Degree, MCC, MNC, and Closeness algorithms as hub genes, which are: Gc, Apoa1, Apoa2, Fgb, Fgg, Fga, Apoh, Hp, Serpina1, Alb, Hrg, F2, Serpinc1, Cpb2, and Plg (Table 3). Based on the *GeneMANIA* plugin in Cytoscape, the protein interaction network of these 15 hub genes was constructed (Figure 5(C)). GO-BP analysis of these 15 hub genes by the *ClueGo* plugin showed that they are mainly associated with acute inflammatory response, coagulation and fibrinolysis, lipid catabolic process, and endothelial cell apoptotic process (Figure 5(D)).

**Table 3.** Twenty-six proteins identified for further validation from differential proteomics analysis of CI-AKI kidney tissues.

Protein Name	Gene	Average Fold Change	*p*-Value	Previously associated with AKI
11 important biomarkers of acute kidney injury that have been reported by reviews [29,30]				
Kidney injury molecule 1	Havcr1/Kim1	3.720	<0.001	Yes
Angiotensinogen	Agt	2.466	<0.001	Yes
Cystatin-C	Cst3	2.230	<0.001	Yes
Liver-type fatty acid-binding protein (L-FABP)	Fabp1	2.143	<0.001	Yes
Dickkopf WNT-signaling pathway inhibitor 3	Dkk3	1.933	<0.001	Yes
Uromodulin	Umod	0.671	<0.001	Yes
Beta-2-microglobulin	Β2m	1.456	<0.001	Yes
Metalloproteinase inhibitor 2	Timp2	1.397	<0.001	Yes
Clusterin	Clu	1.384	<0.01	Yes
Insulin-like growth factor binding protein 7	Igfbp7	1.351	<0.001	Yes
Trefoil factor 3	Tff3	0.534	<0.001	Yes
15 hub proteins of acute kidney injury screened by bioinformatics analysis				
Fibrinogen gamma chain	Fgg	3.559	<0.001	Yes
Fibrinogen beta chain	Fgb	3.391	<0.001	Yes
Albumin	Alb	3.255	<0.001	Yes
Alpha-1-antiproteinase	Serpina1	3.163	<0.001	No
Apolipoprotein A-I	Apoa1	2.852	<0.001	No
Fibrinogen alpha chain	Fga	2.841	<0.001	Yes
Gc-globulin	Gc	2.749	<0.001	Yes [38,39]
Haptoglobin	Hp	2.532	<0.001	Yes [40]
Prothrombin	F2	2.422	<0.001	No
Plasminogen OS	Plg.	2.346	<0.001	No
Apolipoprotein H	Apoh	2.171	<0.001	No
Histidine-rich glycoprotein	Hrg	1.921	<0.001	No
Apolipoprotein A-II	Apoa2	1.730	<0.001	No
Carboxypeptidase B2	Cpb2	1.716	<0.01	No
Antithrombin-III	Serpinc1	1.311	<0.001	Yes

CI-AKI: Contrast-induced acute kidney injury; AKI: Acute kidney injury.

### PRM validation of TMT-based results

**Figure 6.**
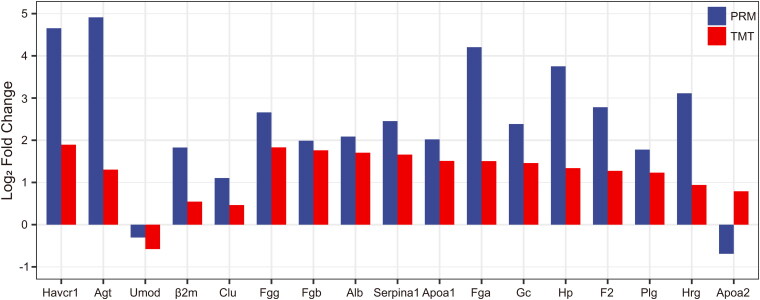
Parallel reaction monitoring outcomes. The abscissa represents the gene name of the protein. The ordinate represents the log2 (Fold change) of the differentially expressed proteins measured by TMT and PRM.

A PRM assay was developed to validate the abundance changes of 26 proteins previously identified by TMT quantitative analysis. There was significant quantitative information for 17 proteins, with Apoa2 trending in the opposite direction of previous TMT proteomics and the other 16 proteins trending in line with previous TMT proteomics: Havcr1, Agt, β2m, Clu, Fgg, Fgb, Alb, Serpina1, Apoa1, Fga, Gc, Hp, F2, Plg and Hrg were upregulated and Umod was downregulated (Figure 6).

## Discussion

CI-AKI is a common clinical complication following iodinated contrast media administration, and its pathogenesis is not fully understood. Effective preventive and therapeutic measures for CI-AKI are lacking. The previous models for studying CI-AKI in rats have certain limitations. For example, the CI-AKI model induced by the L-NAME and indomethacin injection exhibited acute medullary hypoxic injury, which failed to replicate the pathogenic mechanism of CI-AKI [20]. Models pretreated with dehydration to activate the renin-angiotensin system that causes renal vasoconstriction in CI-AKI, including water deprivation and the use of furosemide, showed a mild degree of pathological injury, with changes in serum creatinine that hardly meet the definition of a 26.5 μmol/L increase within 48 h [21,22]. The model developed by 5/6 nephrectomy and dehydration to simulate risk factors for chronic renal insufficiency in CI-AKI patients is complicated and takes much longer to establish [23]. It is generally accepted that chronic renal insufficiency, renal vasoconstriction, and renal medullary hypoxia are the risk factors of CI-AKI, which are simulated in the new model. Our study indicated that dehydration for 24 h only 2 weeks after left nephrectomy, followed by intraperitoneal injection of furosemide and iohexol administration, was sufficient to cause significant changes in serum creatinine and pathological damage to renal tubular cells. In addition, this new model is more stable than the previously reported model of dehydration combined with furosemide administration [21,22] and is easier to manipulate and less time-consuming than the model of partial nephrectomy [23].

Currently, low-osmolality and iso-osmolality contrast agents are commonly used in clinical practice, whereas hypertonic contrast agents are rarely used due to increased nephrotoxicity and high adverse reactions. However, whether iso-osmolality contrast agents are associated with fewer risks for CI-AKI than low-osmolality contrast agents is controversial based on former literature [31–33]. In this study, renal function and pathological alternation caused by iohexol were found to be more severe than those caused by iodixanol in the new model established, suggesting that iohexol is more toxic than iodixanol, which is consistent with other studies [21]. Mitochondria in the iohexol group presented extensive vacuolar degeneration, reduced mitochondrial cristae, and disorganized structure. Mitochondria are intracellular organelles that play key roles in the production of ATP, the primary source and organelle target of ROS, and are of great importance in maintaining cellular homeostasis. Alterations in mitochondrial ultrastructure imply that mitochondrial quality control is involved in the pathogenesis of CI-AKI. Mitochondrial damage and dysfunction can lead to cell death, tissue injury, and possible organ failure [34].

The pathogenesis of CI-AKI remains unclear, and the advent of proteomics provides new approaches to reveal new mechanisms. Although there have been proteomic studies in the urine of CI-AKI patients, few proteomic studies are related to the renal tissue of CI-AKI. In the present study, proteomic analysis of kidney tissues from CI-AKI rats identified differentially expressed proteins, including KIM1, TIMP2, IGFBP7, L-FABP, AGT, UMOD, DKK3, TFF3, and Clusterin, which are good predictors of acute kidney injury in other studies [35,36]. GO enrichment analysis of differentially expressed proteins revealed that in biological processes, wound healing, hemostasis, coagulation, fibrinolysis, and detoxification were enriched; in cellular composition, the apical plasma membrane, extracellular matrix, and hemoglobin complex were enriched; in molecular function, (endo) peptidases and secondary active transmembrane transporter activity were enriched. The KEGG pathway mainly focuses on complement and coagulation cascade, PPAR signaling pathway, mineral absorption, cholesterol metabolism, ferroptosis, and staphylococcus aureus infection.

In this study, we selected 26 proteins (Table 3) for PRM validation, including 15 hub proteins with the strongest interactions in the PPI network and 11 important proteins, and validated 16 proteins with significant differential expressions following the same trend. Five of these 16 proteins were previously reported in AKI-related studies (Havcr1/KIM1, AGT, UMOD, β2M, Clu), 6 were supported by evidence (Fgg, Fgb, Fga, Alb, Gc, Hp), and 5 were new candidate proteins not previously associated with AKI (Serpina1, Apoa1, F2, Plg, Hrg). Fga, Fgb, and Fgg are core genes encoding fibrinogen, which plays an essential role in the negative regulation of endothelial apoptosis and the positive regulation of vasoconstriction, and were significantly upregulated in cisplatin-induced AKI in other research and in the renal tissue of the CI-AKI group in our study [37]. Gc-globulin, also known as Vitamin D-binding protein (VDBP), is an indicator of the response to proximal tubule function [38]. Chaykovska L et al. found urinary VDBP to be a biomarker of the primary clinical outcome within 90 days after contrast exposure [39]. In our experiments, Gc/VDBP was significantly increased in renal tissues of the CI-AKI group. The Hp gene encodes a haptoglobin that captures and binds hemoglobin, allowing the liver to recycle heme and iron and prevent renal damage. Richard A. et al. discovered that Hp protein expression was up to 10 - 100 fold higher in the kidneys of all six AKI mouse models, which was consistent with our proteomic findings [40]. This suggests that Hp gene induction played an important role in the pathogenesis or prognosis of AKI. Serpina1 is a protein that belongs to the serine protease inhibitor family and is thought to be involved in inflammation and coagulation regulation [41]. Apoa1 is a lipid metabolism protein that is a major component of high-density lipoprotein (HDL) [42]. F2 is a coagulation factor family protein that plays a role in blood clotting [43]. Plg is a plasminogen family protein that is involved in the breakdown of blood clots [44]. Hrg is a protein that is involved in the regulation of angiogenesis [45].

There are still some limitations in this research. First, despite the high modeling rate, the CI-AKI rat model established in our study can not fully simulate the pathophysiological process of CI-AKI patients. Second, proteomics technology has such restrictions as insensitivity to small differential proteins, inability to quantify all identified proteins, and the possibility of false positives for detected proteins. Third, the pathways and 16 differentially expressed proteins identified in this study should be further validated, and validation of the current results by measuring blood and urine samples from CI-AKI patients will be our future direction.

## Conclusions

In summary, a rapid and stable CI-AKI rat model based on unilateral nephrectomy was innovatively established in this study. In the new model, the nephrotoxicity of the iso-osmolality contrast agent iodixanol was significantly lower than that of the low-osmolality contrast agent iohexol. By using TMT proteomics and PRM validation, we identified 16 significantly differentially expressed proteins. Five novel proteins not previously reported to be associated with AKI were found as well: Serpina1, Apoa1, F2, Plg, and Hrg, all of which are associated with acute response and fibrinolysis. The pathway analysis and 16 differentially expressed proteins may aid in discovering new mechanisms in the pathogenesis of CI-AKI, allowing for early diagnosis and outcome prediction.

## Institutional review board statement

The study was conducted according to the guidelines of the declaration of Helsinki, and approved by the Animal Ethics Committee of Central South University (No. 2020sydw0899).
